# Sequential Effects of Deep Rolling and Post-Weld Heat Treatment on Surface Integrity of AA7075-T651 Aluminum Alloy Friction Stir Welding

**DOI:** 10.3390/ma12213510

**Published:** 2019-10-25

**Authors:** Adirek Baisukhan, Wasawat Nakkiew

**Affiliations:** 1 Ph.D.’s Degree Program in Industrial Engineering, Advanced Manufacturing Technology Research Center (AMTech), Department of Industrial Engineering, Faculty of Engineering, Chiang Mai University, Chiang Mai 50200, Thailand; adirek_b@cmu.ac.th; 2 Advanced Manufacturing Technology Research Center (AMTech), Department of Industrial Engineering, Faculty of Engineering, Chiang Mai University, Chiang Mai 50200, Thailand

**Keywords:** deep rolling, post-weld heat treatment, fatigue life, surface integrity, friction stir welding, AA7075-T651 aluminum alloy

## Abstract

The aim of this research is to investigate the sequence of processes for improving the welded surface integrity of AA7075-T651 aluminum alloy joined by friction stir welding (FSW). The improvement processes that will be investigated herein include mechanical surface improvement with deep rolling (DR) and post-weld heat treatment (PWHT). Therefore, this study investigated welded surface integrity, which comprises residual stress, microhardness, surface roughness, microstructure, and fatigue life (screening). The experiment consists of three sets of combinations. In the first set, only FSW was applied; in the second set, FSW was applied, followed by DR, and then PWHT processes (FSW-DR-PWHT); and in the last set, FSW was applied, followed by PWHT, and then DR processes (FSW-PWHT-DR). Fatigue testing was carried out by undertaking a four-point bending test using a bending stress of approximately 300 MPa with a test frequency of 2.5 Hz at room temperature and stress ratio R = 0. The study found that residual stress plays an important role in the fatigue life. Finally, the fatigue test showed that FSW workpieces subject to the PWHT process followed by the DR process (FSW-PWHT-DR) had the highest fatigue life, with an increase of 239% when compared with unprocessed FSW workpieces.

## 1. Introduction

Friction stir welding (FSW) is a popular aluminum welding process that can also be used to weld dissimilar materials. It was invented in 1991 in the United Kingdom by The Welding Institute (TWI). The heat involved in FSW is generated by friction between the shoulder and the pin of a rotating nonconsumable welding tool and the workpiece, which is firmly fixed throughout the FSW process. As a welding process, FSW does not require an external heat source or the addition of a welding wire filler [1]. The FSW process involves several welding parameters, including rotational speed, feed rate, tilt angle, penetration depth, characteristics of the welding tool used (pin length, pin characteristics, shoulder diameter, and shoulder shape), material properties, cooling system, clamping system, etc. These factors affect the quality of the weld and must be controlled if the materials involved are to be properly welded [2,3,4]. Residual stresses follow the FSW process due to the nonuniform cooling rate of the welding [5,6], mainly tensile residual stress, which affects the fatigue strength and fatigue life of the welded workpiece. In general, the mechanism of fatigue fracture depends on the level of the stress or load used. In the case of low-cycle fatigue with a high-stress level, the fracture area consists of two zones: ductile fracture and brittle fracture [7,8]. Furthermore, the near-surface stress concentration and the friction stir welded joint defect such as voids in the bottom of the welded joint, kissing bonds cracks, root sticking, weld root flaw, etc. will shorten the fatigue life [9]. Therefore, deep-rolling and post-weld heat treatment processes are used to improve FSW workpieces, making them stronger and giving them a longer fatigue life. Many mechanical surface improvement processes can be used, such as shot peening (SP), deep rolling (DR), laser shock peening (LSP), etc. For those workpieces that need improvement, uncomplicated procedures, and good results, the DR process can be used and provides a particularly suitable option. The DR process can cause the near-surface area to experience compressive residual stress due to the pressure of the roller or sphere (Hertzian pressure), resulting in a smooth surface. When the DR force is greater than the yield strength of the material, at the near-surface, local plastic deformations occur as well as work hardening effects and compressive residual stress in order to increase fatigue strength [10,11,12]. Process parameters related to the DR process include force or pressure, feed rate, contact length, number of overruns, rolling direction, etc. These factors affect results and need to be controlled correctly and appropriately. Compressive residual stress and work hardening near the surface of the workpiece play important roles in preventing or reducing the initiation and growth of fatigue cracks [13,14,15,16]. Consequently, FSW workpieces that have undergone the improvement processes have a longer service life. Post-weld heat treatment (PWHT) is a post-welding process whereby welded parts are heated again using lower temperatures than the critical transformation temperature; the workpiece is soaked at these temperatures over a specified period. Soaking time, temperature, and cooling rate are important, and such factors must be controlled to prevent unwanted results. It is well known that welding causes residual stress within workpieces. The PWHT process reduces the residual stress to an acceptable level while also improves the hardness, strength, toughness, and ductility of the workpiece to higher levels [17,18,19,20,21]. The PWHT process of solution and artificial aging (STA) provides the most advantageous results compared with other methods, allowing better tensile strength and improved hardness to higher values for FSW workpieces [17]. The concept of surface integrity was presented by M. Field and J. Kahles in 1964, and they characterized surface integrity as the improved condition of a surface delivered in manufacturing surface operation, which also includes the welding process. Surface integrity includes all of the components that describe all conditions of the existing surface. It takes care of not the surface topography but the metallurgical character of the surface and subsurface. Each surface modification handle is related to the change of different properties counting residual stress, surface roughness, plastic deformation, microhardness, micro-cracking, phase transformations, fatigue life, etc. [22,23]. Surface integrity is the evaluation of the effect of FSW on the surface properties of the workpiece.

The literature does not contain research concerned with two improvement processes, DR and PWHT, used together for an AA7075-T651 aluminum alloy friction stir welded joint to improve its surface integrity and fatigue life (screening). The aim of this research is to investigate the sequence of these processes to improve the welded surface integrity of FSW workpieces using DR and PWHT processes on an AA7075-T651 aluminum alloy. The effects of these improvement processes on microhardness, microstructure, surface roughness, residual stresses—together with work hardening characterized by the full width at half maximum (FWHM)—and screening of fatigue life are investigated and discussed.

## 2. Materials and Methods 

The material used in this research is the AA7075-T651 aluminum alloy. The chemical composition values for this material (all units in % *w*/*w*), as measured by the energy-dispersive X-ray fluorescence (EDXRF) method (model JSX3400R, JEOL, Tokyo, Japan), and mechanical properties are as shown in Table 1 and Table 2, respectively [24].

**Figure 1:**
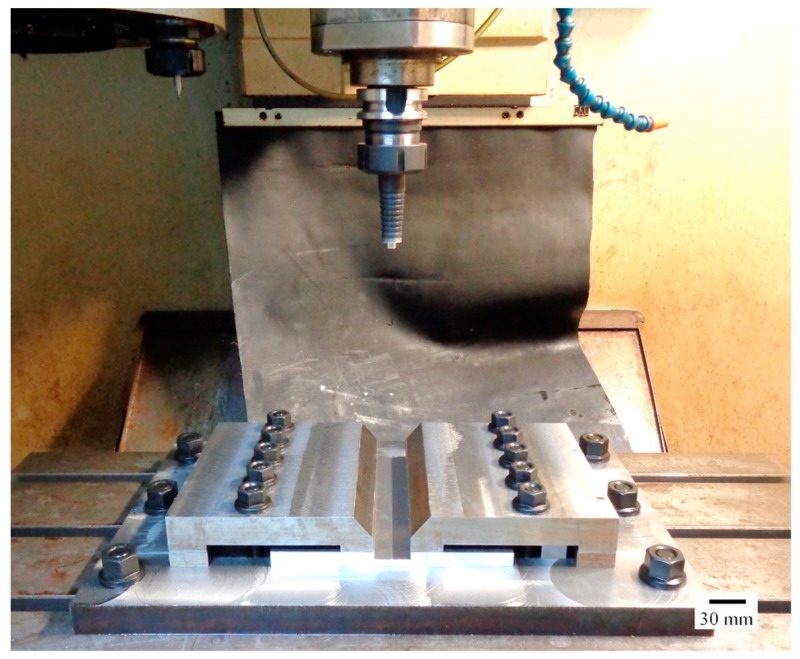
Experimental setup of friction stir welding on a Computer Numerical Control (CNC) machine.

The dimensions of the workpiece used in this experiment are as follows: 100-mm width, 200-mm length, and 6.5-mm thickness; two sheets were used per experiment. The workpiece is securely fastened to the mechanical clamping device to prevent it from moving during the FSW process. FSW was done using the Bridgeport Computer Numerical Control (CNC) machine, model VMC500, as shown in Figure 1. The parameters used for the FSW process [25] are shown in Table 3.

**Figure 2:**
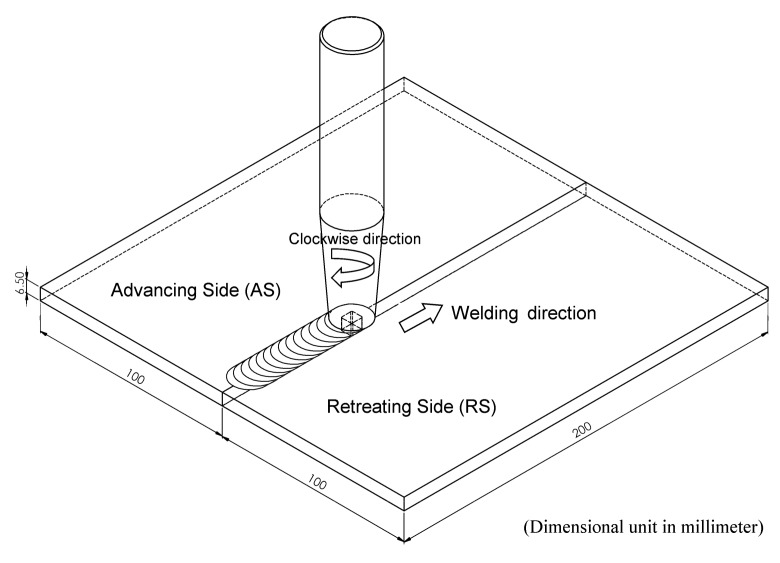
Schematic diagram of the friction stir welding (FSW) process.

**Figure 3:**
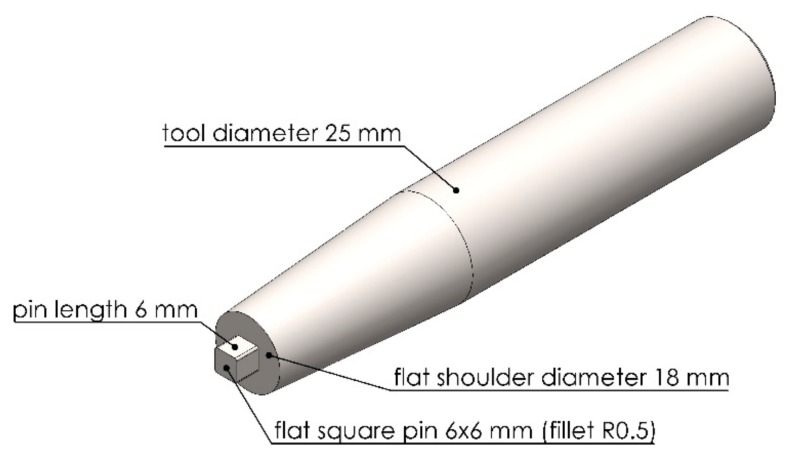
FSW tool geometry and dimensions.

**Figure 4:**
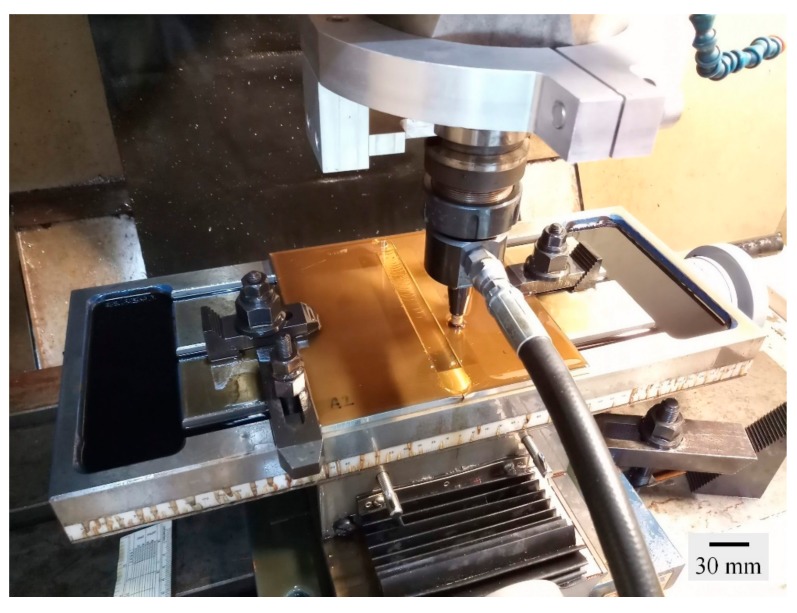
FSW workpiece during the deep-rolling (DR) process.

**Figure 5:**
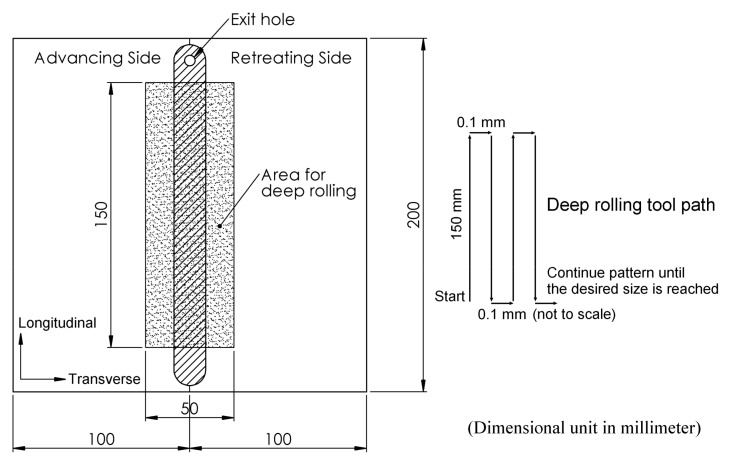
Designed area and tool path for the deep-rolling process.

In this FSW, the welding tool is rotated in a clockwise direction and welding from the bottom edge to the top edge of the workpiece is carried out. Therefore, the left side and the right side of the welding tool are called the advancing side (AS) and retreating side (RS), respectively, as shown in Figure 2. A square butt joint was used with single-pass welding with a cycle time of 465 s per cycle. Concerning the welding tool head profile, a flat square pin sized 6 × 6 mm with a 6-mm pin length and 18 mm of flat shoulder diameter was used, as shown in Figure 3. The nonconsumable welding tools used were made from Japanese Industrial Standards (JIS) SKD61 material, equivalent to American Iron and Steel Institute (AISI) H13 or Deutsches Institut für Normung e.V. (DIN; German Institute for Standardization) 1.2344, with a hardness of 55 HRC after they had been hardened by the vacuum heating process followed by nitrogen quenching. The FSW workpieces were then subject to DR and PWHT processes, according to the individual experimental sequences. An Ecoroll hydrostatic tool, model HG6, was used for the DR process, which is a surface-improvement process. The hydraulic oil Total Lactuca LT3000 was used without mixing to provide the required pressure to the DR tool. The DR process was also undertaken using the Bridgeport CNC, model VMC500. The FSW workpiece was securely fastened to the mechanical clamping device to prevent the workpiece from moving during the DR process, as shown in Figure 4. The conditions used for the DR process [25] are shown in Table 4. The area for deep rolling is 50 mm in width and 150 mm in length and the tool path of the deep-rolling process starts from the lower left corner of the designated area, as shown in Figure 5. The deep-rolling tool will move in the longitudinal direction to the end of welded area for a distance of 150 mm, then move to the right in the transverse direction to a distance of 0.1 mm, and then move to the origin of the welded area in the longitudinal direction for a distance of 150 mm. The deep-rolling tool then moves to the right in the transverse direction to a distance of 0.1 mm and then moves in the longitudinal direction to the end of welded area for a distance of 150 mm, continuously moving in this pattern until achieving the area as designed. Each workpiece is deep rolled for one round.

**Figure 6:**
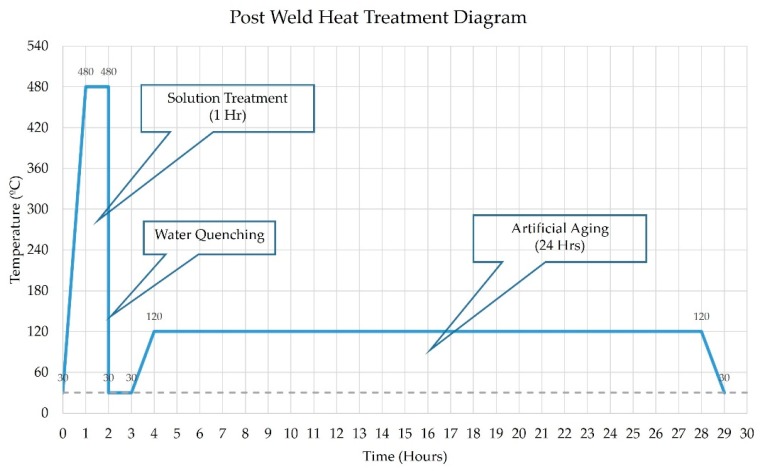
Post-weld heat treatment (PWHT) diagram.

The PWHT process was undertaken to relax the residual stress that occurs after welding and, also, to improve the material’s mechanical properties at the design level. The effect of the PWHT process on the AA7075-T651 aluminum alloy FSW workpiece revealed that solution treatment (480 °C soaking for 1 h) followed by quenching with water and, then, an artificial aging cycle (120 °C soaking for 24 h), as shown in Figure 6, had the benefit of increasing the material’s tensile strength and hardness [17]. Accordingly, the conditions of such PWHT processes were used in this research.

Aging temperature and aging time have an effect on residual stresses, including the value of FWHM, which will reduce during an aging time; all of the above will be controlled by thermally activated relaxation processes that can be described by a Zener–Wert–Avrami function, as shown in Equation (1):

(1) $$\sigma^{rs}/\sigma_{0}^{rs}=\exp[-{\left(At_{a}\right)}^{m}]$$

where

$\sigma^{rs}$ = an residual stress under aging temperature $T_{a}$ and aging time $t_{a}$
$\sigma_{0}^{rs}$ = an initial residual stress
$t_{a}$ = an aging time
m = a numerical term dependent on the dominant relaxation mechanism
     (m value between 0.1 to 0.3 for non-ferrous alloy)
A = a function dependent on the material and temperature according to Equation (2):

(2) $$A=B\exp(-\Delta H/kT_{a})$$

where

B = a material constant
k = a Boltzmann constant
ΔH = the activation enthalpy for the relaxation process
$T_{a}$ = an aging temperature

From Equation (1), a plot of log ln $\left(\sigma_{0}^{rs}/\sigma^{rs}\right)$ as a function of log $t_{a}$ for a constant aging temperature $T_{a}$ gives a straight line of slope m. $\sigma^{rs}$ and $\sigma_{0}^{rs}$ can be obtained from the measured residual stress data during artificial aging according to time and temperature. Moreover, from Equation (2), ΔH can be determined by fitting the slope of the plot between log$t_{a}$ and $1/kT_{a}$ [26,27,28].

As part of this research, each workpiece will undergo various processes. It can be concluded that three workpiece combinations should be considered prior to analyzing fatigue life and surface integrity so that the efficacy of each can be compared.

1. For the first workpiece, only FSW was applied.
2. For the second workpiece, FSW was applied, followed by DR, and then PWHT processes (FSW-DR-PWHT).
3. For the third workpiece, FSW was applied, followed by PWHT, and then DR processes (FSW-PWHT-DR).

**Figure 7:**
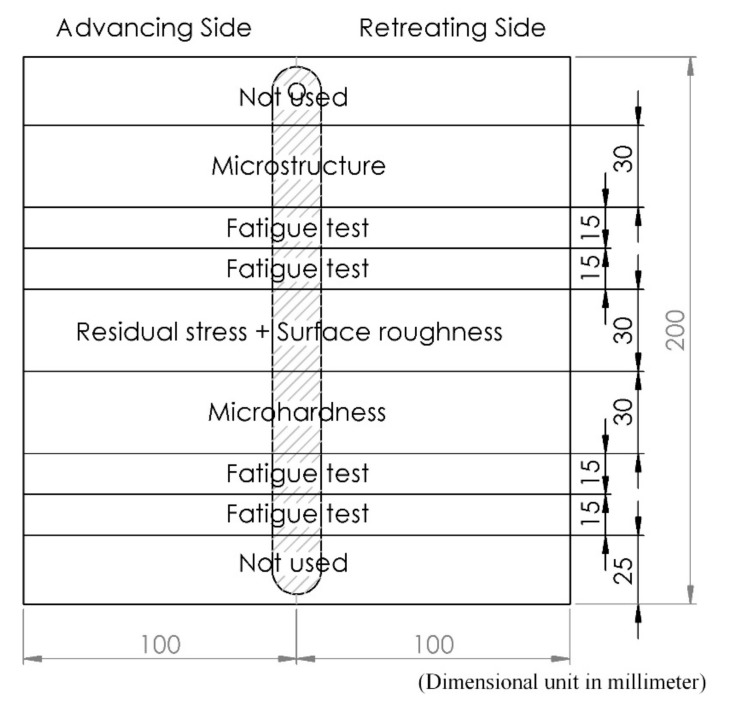
Each part of the workpiece.

The surface integrity study in this manuscript is performed in five steps (residual stress, microhardness, surface roughness, microstructure, and fatigue life), and the workpiece is divided into parts, as shown in Figure 7. The workpiece was cut with a band saw that has a coolant while cutting and using a slow cutting speed.

**Figure 8:**
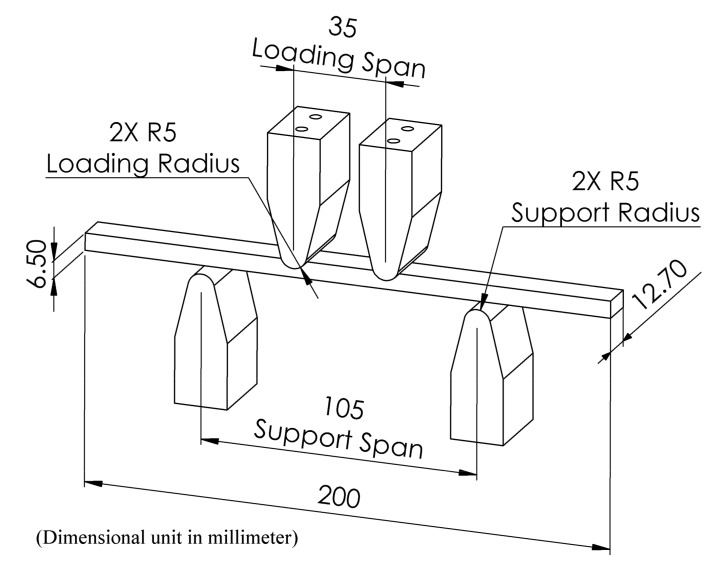
Test workpiece detail of American Society for Testing and Materials (ASTM) D6272.

Fatigue testing of the FSW workpieces was carried out by undertaking a four-point bending test using a bending stress of approximately 300 MPa, equivalent to a total force of 1545 N. From the literature, it was found that, in most cases, FSW joints have about 70% joint efficiency compared to parent materials. Thus, the ultimate tensile strength and yield strength of the AA7075-T651 aluminum alloy will be approximately 394 MPa and 335 MPa, respectively. Therefore, the fatigue test should be tested at less than 10% of yield strength, resulting in the bending stress for a fatigue test at 300 MPa. The fatigue testing machine was equipped with the programmable logic controller (PLC) system to control the pneumatic devices through the human machine interface (HMI) screen. Tension–tension fatigue tests were conducted with a pneumatic system using a test frequency of 2.5 Hz at room temperature and stress ratio R = 0. The workpiece and fixture dimensions used accorded with American Society for Testing and Materials (ASTM) D6272, as shown in Figure 8. The dimensions of the workpiece are 12.7 mm wide, 200 mm in length, and 6.5 mm in thickness, while the workpiece uses the support span at 105 mm (support span to thickness ratio about 16:1), and the loading span will be 1/3 of the support span at a distance of 35 mm. Place the weld seam of the workpieces face down on the support span as the load will cause tension on the surface welded joint. Four workpieces were tested to determine the fatigue life, and then, the average value of these tested were calculated. Formulae for calculating the four-point bending test, where the loading span is 1/3 of the support span with a rectangular cross-sectional area, are shown in Equation (3):

(3) $$\sigma =\frac{FL}{bd^{2}}$$

where

σ = Bending stress at outer side (MPa)
*F* = Load at defined point on the load deflection curve (N)
*L* = Length of support span (mm)
*b* = Width of test workpiece (mm)
*d* = Thickness of test workpiece (mm)

**Figure 9:**
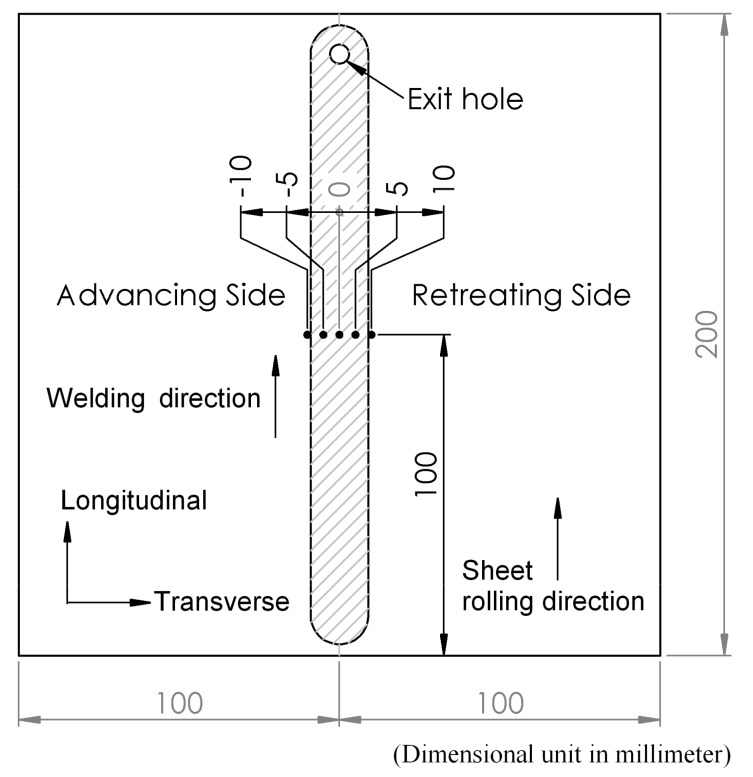
Position for measuring residual stress.

Residual stress of the FSW workpieces will be measured using X-ray diffraction (XRD) using the sin^2^ψ method, which is a nondestructive method. Residual stresses are decided from the diffraction information by calculating the strain from the diffraction peak positions. Stresses induce a strain, which compares to changes in lattice spacing. Residual stresses at that point are calculated by measuring lattice spacing with numerous tilt angles and by plotting the results about a d versus sin²ψ chart, where ψ is the tilt angles and d is the measured lattice distance. The residual stresses can be decided from the slope of this d versus sin²ψ chart, together with the modulus of elasticity and Poisson number of the material, which are used to determine the residual stresses that occur. The width of the diffracted peak is measured as full width at half maximum (FWHM), which was affected by micro-stresses and/or hardness and imperfections in the crystal structure, such as plastic deformation, dislocations, etc. Typically, the values of these properties increase with increasing hardness [29]. The Stresstech Group’s XSTRESS 3000 model equipped with a Chromium tube source radiation was used for this purpose. Eleven positive- and negative-value tilt angles were used (0, 18.4°, 26.6°, 33.2°, 39.2°, and 45°) and the temperature during measurement was about 25 °C. Residual stresses were measured in the longitudinal direction using XRD at the following distances from the welding center line: −10, −5, 0, 5, and 10 mm, comprising a total of five points for each workpiece, as shown in Figure 9. 

**Figure 10:**
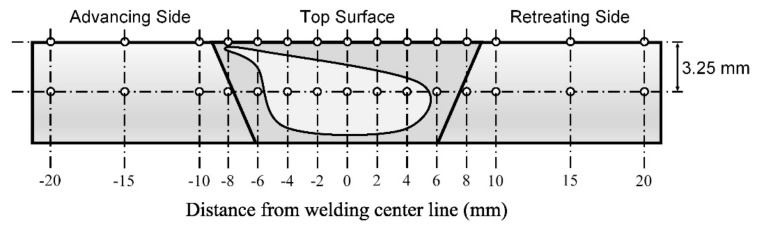
Position for measuring microhardness.

Vickers microhardness tests were undertaken using Anton–Paar’s MHT-10 model with an applied force of about 0.4903 N or HV0.05, with a 1 g/s load speed, a 15 s holding time, and objective 50×. The cross-sectional area of the workpiece was prepared by measuring the hardness from the center of the FSW (measured at a distance below the surface about 3.25 mm) from left to right and by measuring the surface of the workpiece itself, as shown in Figure 10. In total, 15 positions were measured, with each position being 2 mm apart except for last two positions, which were 5 mm apart. Each position was measured twice; an average of these measurements was then calculated and graphically displayed. 

Surface roughness was tested using the Mitutoyo’s Surftest SJ-400. The general surface parameters, which are employed in this research, are R_a_ (arithmetical mean roughness value) and R_t_ (total height of the roughness profile). Each workpiece was measured three times, and an average of these measurements was calculated. To apply optical characterization to the FSW workpieces, each workpiece was prepared by being cut into smaller areas. The workpiece was cut with a band saw using coolant and a slow cutting speed. Consequently, the cut workpieces were small and difficult to hold while grinding and polishing, and so, each was placed in a resin mold made by a hot mounting machine. The workpieces were cylindrical with a diameter of 25 mm and were ground and polished to create a smooth surface. For the etching process, the surface of the workpiece was prepared using a chemical corrosive to remove oxide inclusions within the microstructure. Keller’s reagent was used [30], which comprises 190 mL of deionized water, 5 mL of nitric acid (HNO_3_), 3 mL of hydrochloric acid (HCl), and 2 mL of hydrofluoric acid 48% (HF). The workpieces were then immersed for about 15 s before being cleaned using warm water and blow dried. The Keller’s reagent was replaced for each workpiece. Microstructural pictures of the FSW workpieces were taken using an optical microscope at 50× and 500× magnification.

## 3. Results and Discussion

### 3.1. Residual Stress

**Figure 11:**
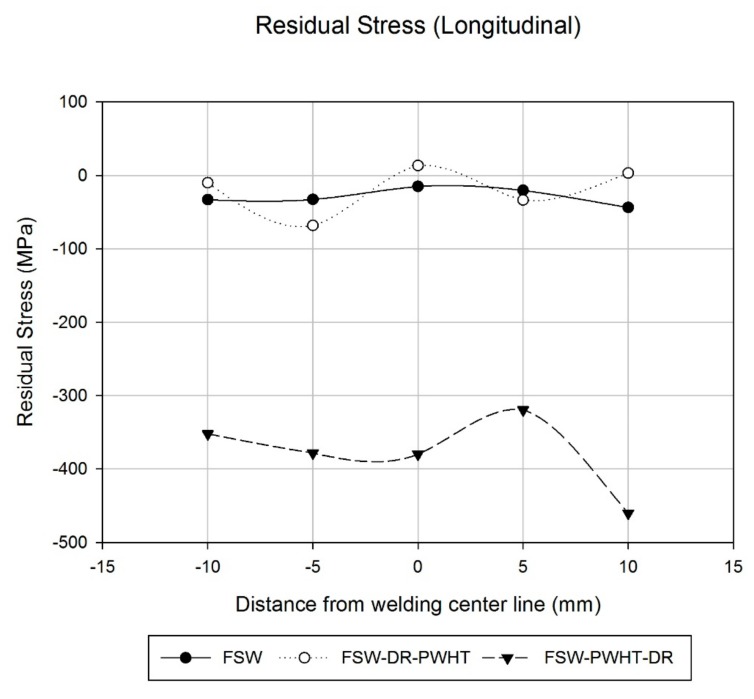
Residual stress in longitudinal direction.

**Figure 12:**
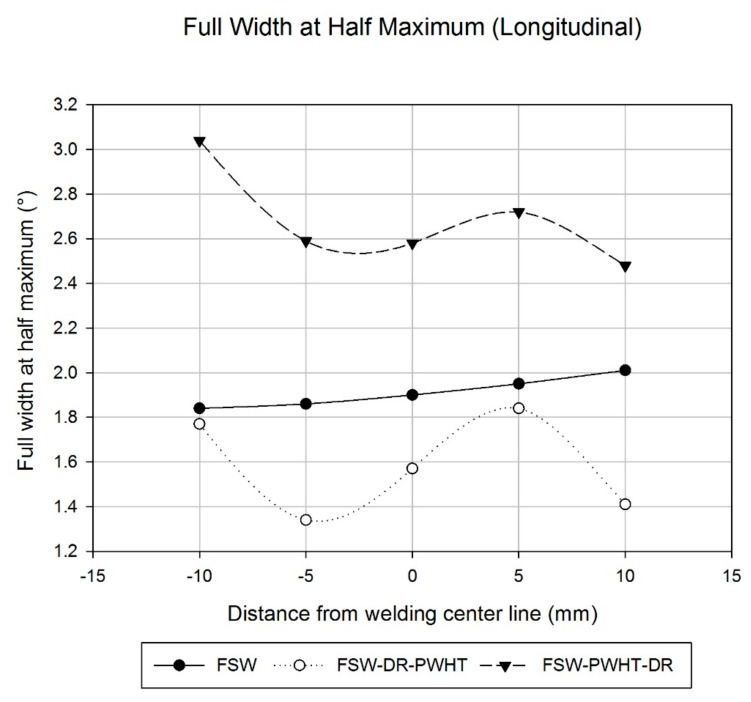
Full width at half maximum value in the longitudinal direction.

The FSW workpiece showed a uniform surface residual stress throughout the welding area, with values of the advancing side (AS) being about −33 MPa, the stir zone (SZ) at about −15 MPa, and retreating side (RS) having a value of −20.3 MPa reduced to −43.6 MPa at the 5-mm and 10-mm positions from the welding center line, as shown in Figure 11. Accordingly, the FSW workpiece, when subject to the welding conditions investigated in this research, was found to be satisfactory according to the compressive residual stress results. Comparatively, the surface value of the FWHM work hardening state [10,11] was found to increase slightly from the AS to the RS from 1.84° to 2.01°, respectively, as shown in Figure 12.

Concerning the FSW-DR-PWHT workpiece, it was revealed that the surface residual stress value of the AS decreased from −9.9 MPa to −68.1 MPa at positions −10 mm and −5 mm from the welding center line, respectively. The corresponding SZ was about 13.5 MPa, and the RS value was found to increase from −33.5 MPa to 3.2 MPa at positions 5 mm and 10 mm from the welding center line, respectively. The surface residual stress value obtained concerns both tensile residual stress and compressive residual stress of the welding area as well as the FWHM value at positions −5 mm and 5 mm from the welding center line, which revealed a lowest value of about 1.34° and a highest value of about 1.84°. Therefore, the FSW-DR-PWHT workpiece is expected to have a shorter fatigue life than that of the FSW workpiece due to the last treatment process it underwent: the PWHT process, which caused residual stress and FWHM to relax after artificial aging treatment. The results are consistent with the reference [10,18]. Comparatively, the FSW-PWHT-DR workpiece revealed a reduced surface residual stress at AS, from −352.1 MPa to −378.2 MPa at positions −10 mm and −5 mm from the welding center line, respectively, with a SZ of about −379.8 MPa; at the RS, the value was reduced from −319.4 MPa to −460.2 MPa at positions 5 mm and 10 mm, respectively, from the welding center line.

All measured values of the FSW-PWHT-DR workpiece concern compressive residual stress and were found to be significantly different from those of other workpieces, while the FWHM values were slightly reduced from the AS to the RS from 3.04° to 2.48°, respectively. Both compressive residual stress and FWHM values of the FSW-PWHT-DR workpiece were the most measured values as compared with those of other workpieces. Therefore, from the residual stress results and the FWHM values, it can be predicted that the FSW-PWHT-DR workpiece will provide the greatest fatigue life when compared with those of other workpieces concerned herein.

### 3.2. Microhardness

**Figure 13:**
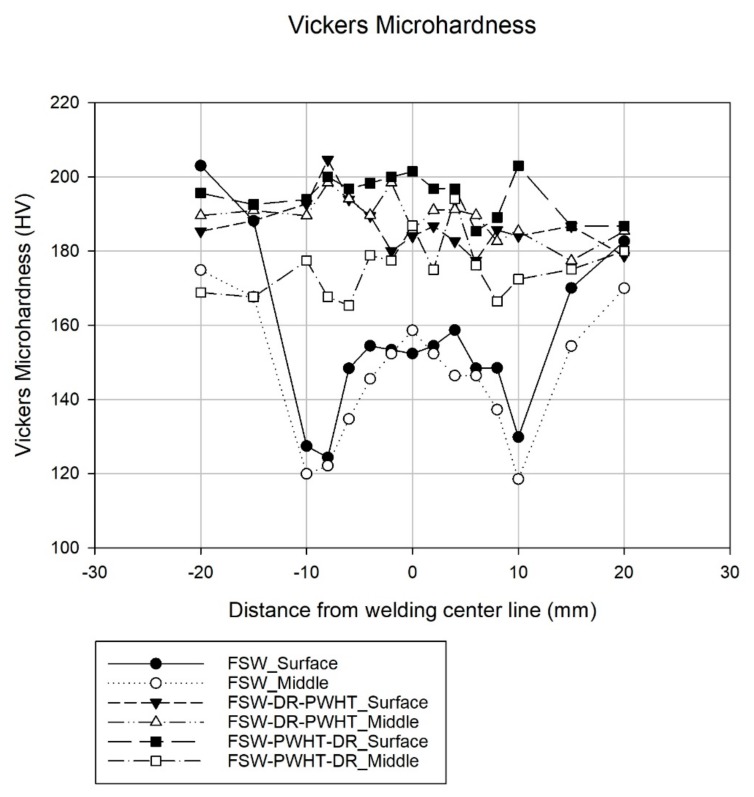
Vickers microhardness test results.

All workpieces in the base material at the position of −20, −15, 15, and 20 mm from the welding center line can be considered as having similar values, with a microhardness of approximately 185 HV, as shown in Figure 13. The SZ of the FSW workpiece, both at the surface and at the middle of the workpiece itself, had a uniform microhardness of approximately 150 HV, which is lower than that of the base material. The lowest microhardness point of the FSW workpiece was located in the thermo-mechanically affected zone (TMAZ) both on the AS and the RS, with a microhardness of approximately 125 HV. It was found that, for the FSW-DR-PWHT workpiece, microhardness was almost uniformly consistent throughout the surface and the middle of the workpiece, with a microhardness of approximately 188 HV, and similar results were reported elsewhere [3]. This is was the highest recorded microhardness among all the workpieces concerning this middle area. The microhardness of the FSW-PWHT-DR workpiece was found to be approximately 195 HV almost uniformly throughout the surface, the highest microhardness found among all the workpiece surface areas. This is because the DR process is the last step in surface improvement, resulting in the near- surface area of the workpiece FSW-PWHT-DR having the highest surface microhardness due to local plastic deformations, resulting in work hardening. The microhardness value of FSW-PWHT-DR at the middle of the workpiece was found to have the same consistency, with an average microhardness of 175 HV, which was a lower microhardness than that of the surface of the workpiece.

When comparing the microhardness at the surface of the workpiece, it was found that the microhardness of the FSW-PWHT-DR workpiece increased by 5.4% compared with that of the base material and that the microhardness increased by 30% compared with that of the welded joint at SZ of the FSW workpiece.

### 3.3. Surface Roughness

**Figure 14:**
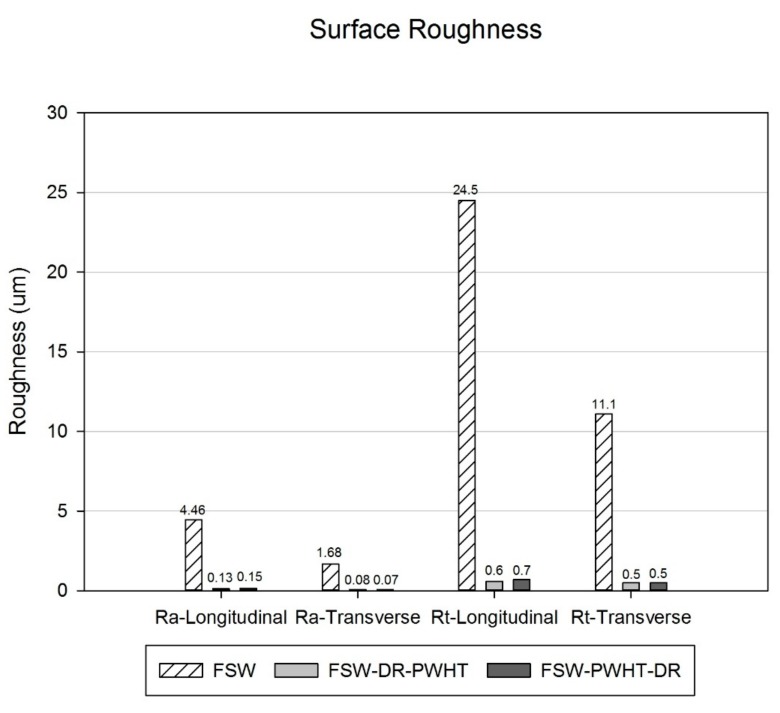
Surface roughness test results.

Surface roughness results are shown in Figure 14. It was found that the R_a_ value decreased in the longitudinal direction from 4.46 µm to 0.13 µm and 0.15 µm and from 1.68 µm to 0.08 µm and 0.07 µm in the transverse direction concerning the FSW, FSW-DR-PWHT, and FSW-PWHT-DR workpieces, respectively. The R_t_ value revealed the same tendencies as that of the R_a_ value, which decreased from 24.5 µm to 0.6 µm and 0.7 µm in the longitudinal direction as well as from 11.1 µm to 0.5 µm and 0.5 µm in the transverse direction for the FSW, FSW-DR-PWHT, and FSW-PWHT-DR workpieces, respectively. In comparison, it was found that the FSW-DR-PWHT and FSW-PWHT-DR workpieces had a similar surface roughness value, which can be considered as being equal. Furthermore, the surface roughness of the longitudinal direction was found to be close to that of the transverse direction due to the pressure involved in the DR process, which makes the surface smooth and consistent. Both of these workpieces had to undergo the DR process, although the sequence in which the DR and PWHT processes were applied differed. In comparison, it was found that the FSW workpiece that did not undergo the DR process had a noticeably greater roughness than the workpiece that did undergo the DR process. Accordingly, it can be concluded that the DR process improved the surface (decreased the value of surface roughness) of the FSW workpiece to a level equivalent to that of the surface roughness of the grinding process.

### 3.4. Microstructure

**Figure 15:**
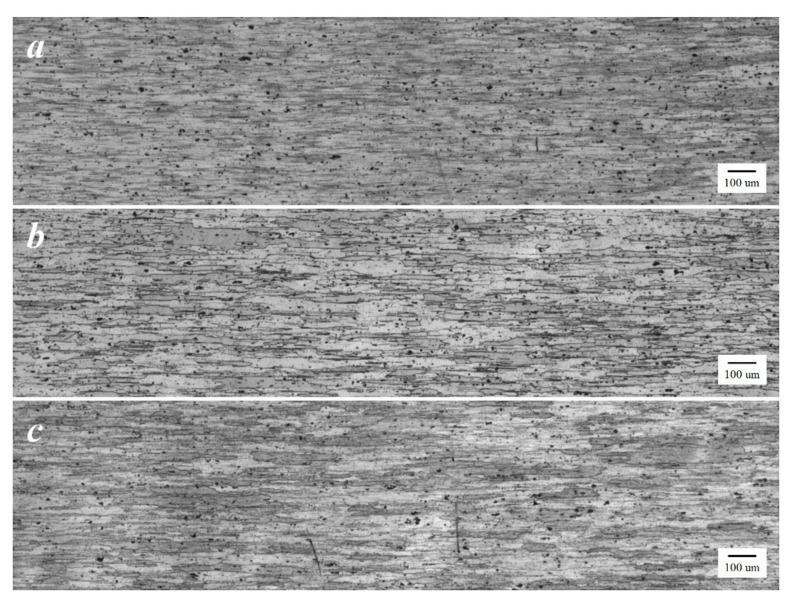
Microstructure of base material (BM) at 50× (**a**) FSW; (**b**) FSW-DR-PWHT; and (**c**) FSW-PWHT-DR.

**Figure 16:**
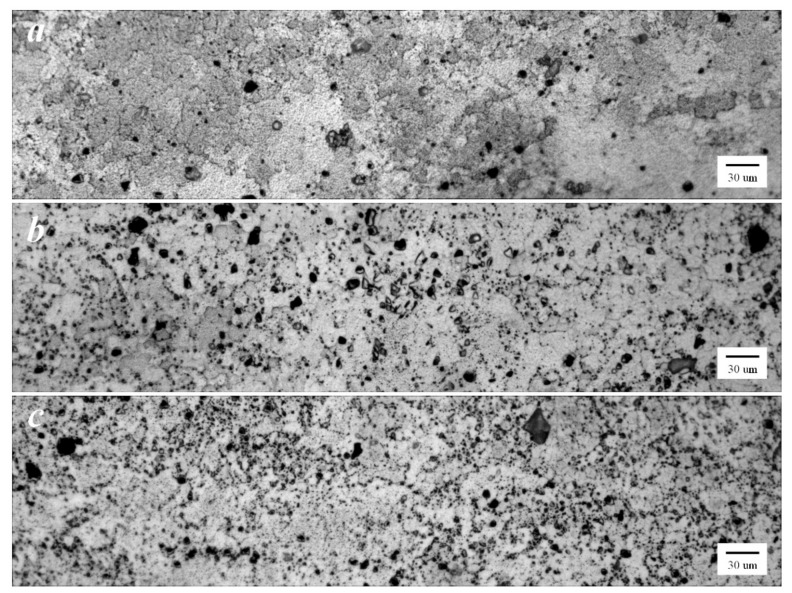
Microstructure of the stir zone (SZ) at 500× (**a**) FSW; (**b**) FSW-DR-PWHT; and (**c**) FSW-PWHT-DR.

**Figure 17:**
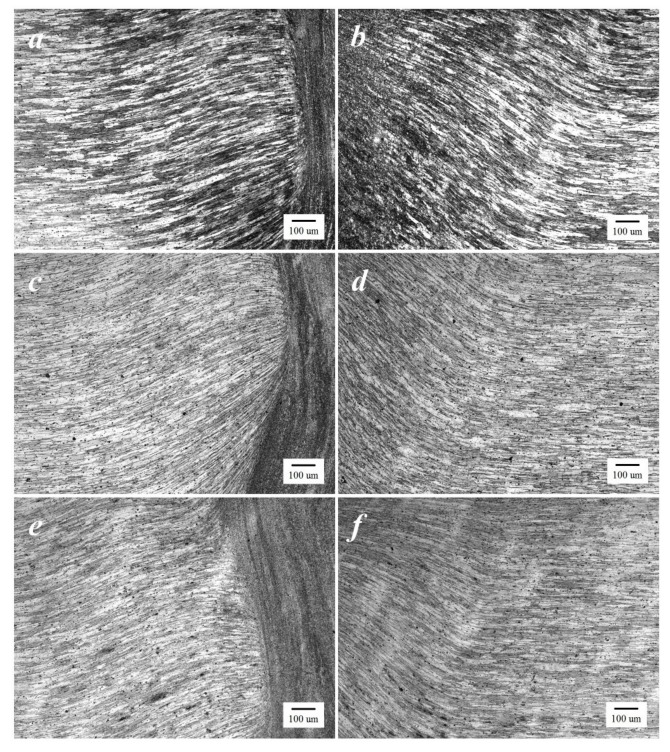
Microstructure at 50× (**a**) thermo-mechanically affected zone (TMAZ)-advancing side (AS) of FSW; (**b**) TMAZ-retreating side (RS) of FSW; (**c**) TMAZ-AS of FSW-DR-PWHT; (**d**) TMAZ-RS of FSW-DR-PWHT; (**e**) TMAZ-AS of FSW-PWHT-DR; and (**f**) TMAZ-RS of FSW-PWHT-DR.

The optical micrograph of the base material (BM) of the FSW workpieces as well as those subjected to FSW-DR-PWHT and FSW-PWHT-DR sequential processes are shown in Figure 15a–c, respectively. These workpieces exhibited similar average grain structures and similar distributions of precipitates. The SZ of the FSW workpiece, as shown in Figure 16a, exhibited an equal and fine-grain size due to continuous dynamic recrystallization (CDRX) during welding. Additionally, the SZ of FSW-DR-PWHT and FSW-PWHT-DR workpieces, as shown in Figure 16b,c, respectively, exhibited a marginal reduction in grain size due to the PWHT process. The TMAZ-AS and TMAZ-RS of the FSW workpiece are shown in Figure 17a,b, respectively. This workpiece exhibited an elongated, bent, narrow, and relatively coarse grain structure compared with that of SZ due to the temperature and strain being inadequate for recrystallization. Moreover, the TMAZ-AS and TMAZ-RS of the FSW-DR-PWHT and FSW-PWHT-DR workpieces, as shown in Figure 17c–f, respectively, exhibited smaller grain sizes and greater distributions of precipitates compared with those of the FSW workpiece. This result is attributable to the PWHT process.

### 3.5. Fatigue Life

**Figure 18:**
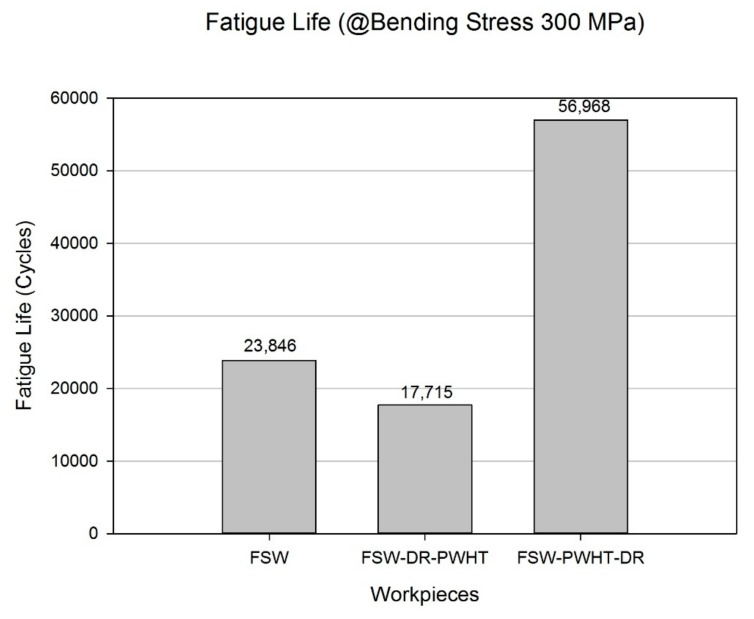
Fatigue life of the friction stir welded workpiece.

**Figure 19:**
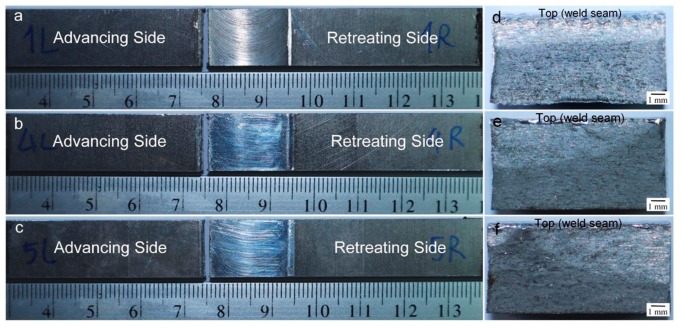
Photographs of the broken workpiece from fatigue testing (**a**) FSW; (**b**) FSW-DR-PWHT; (**c**) FSW-PWHT-DR; (**d**) cross section of FSW (advancing side); (**e**) cross section of FSW-DR-PWHT (advancing side); and (**f**) cross section of FSW-PWHT-DR (advancing side).

Results of the experiments are shown in Figure 18. The FSW workpiece was found to have a fatigue life of 23,846 cycles. Comparatively, the FSW-DR-PWHT workpiece was found to have a fatigue life of 17,715 cycles, corresponding to 74% of the FSW workpiece fatigue life, while the FSW-PWHT-DR workpiece was found to have a fatigue life of 56,968 cycles, corresponding to 239% of that of the FSW workpiece fatigue life. All these values pertain to the TMAZ-AS area at which each workpiece was broken, as shown in Figure 19a–c. From Figure 19d–f, it was found that the cross-sectional area of broken workpieces constituted two main areas: the white area and the dark area; the white area is the area where fatigue crack propagation occurs. The surface of the white area has more flatness than the dark area. White areas can also be called the fatigue zone or beach marks. The dark area is the area where a fast fracture or overload occurs; the surface of this area is rough. Figure 19f has the most white areas compared to the white areas of Figure 19d,e. This shows that the workpiece of Figure 19f, which is the FSW-PWHT-DR workpiece, has more fatigue life than other workpieces. The FSW-PWHT-DR workpiece had the highest fatigue life due to it having the highest compressive residual stress at the surface compared with those of other workpieces, and similar results were reported [14]. Tensile loads were performed in a cycle, causing damage to the workpiece. Accordingly, when compressive residual stress remains on the work surface, the two forces are offset by one another, resulting in increased fatigue life. Concerning surface hardness, it cannot be clearly indicated whether the workpiece has a greater fatigue life as the result of residual stress. However, the trend suggests that, if the surface hardness was high, the fatigue life tended to be higher.

The FSW workpiece was found to have compressive residual stress around the welded area, which differs from conventional fusion welding, where the welded area has a tensile residual stress that results in FSW workpiece resistance to fatigue load at a certain level but not to the same degree as that seen in the FSW-PWHT-DR workpiece. The FSW-DR-PWHT workpiece was found to have the lowest fatigue life due to the last treatment process it underwent, the PWHT process, which caused residual stress to relax during aging treatment, and similar results were reported elsewhere [3,10]. This is explained by the Zener–Wert–Avrami function. 

## 4. Conclusions

Investigation of the sequence of DR and PWHT processes to improve the welded surface integrity of FSW, FSW-DR-PWHT, and FSW-PWHT-DR workpieces on AA7075-T651 aluminum alloy led to the following conclusions:

1. Measured values were established by the last step of the treatment. It was found that the greatest benefit concerning both treatment process sequences was yielded when the workpiece was first subject to the PWHT process followed by the DR process (FSW-PWHT-DR).
2. The FSW-PWHT-DR workpiece can enhance the fatigue life of the material by up to 239% when compared with FSW workpieces that have not undergone any improvement processes. Comparatively, it was found that the FSW-DR-PWHT workpiece resulted in the fatigue life of the material being reduced by up to 26% when compared with the FSW as-welded workpiece. The results of the DR process, the last step of treatment, are that the DR process can enhance the fatigue life.
3. Near-surface compressive residual stress has confirmed that a great influence on fatigue life of the welded joint due to the near-surface compressive residual stress can prevent or reduce fatigue surface crack initiation as well as surface fatigue crack growth.
4. The DR process improves the surface roughness (decreases the value of surface roughness) to a level equivalent to that of the grinding process with the R_a_ values in the longitudinal and transverse directions of the FSW-PWHT-DR workpiece at 0.15 µm and 0.07 µm, respectively. Comparatively, the PWHT process relaxes near-surface residual stress during aging treatments but will increase the hardness value as well as improve the hardness uniformity.

This research found that the sequences of two improvement processes, the DR process and the PWHT process, that apply to AA7075-T651 aluminum alloy FSW joints affect the surface integrity which consists of residual stress, microstructure, surface roughness, microhardness, and fatigue testing (screening). Therefore, the sequences of two improvement processes should be used appropriately to maximize the benefits of the FSW workpiece. In the future, additional fatigue testing will be performed to obtain the S–N graph in order to compare stress values with the number of cycles of each workpiece. Since this manuscript has only reported fatigue testing screening, in general, fatigue testing requires testing at different stress levels (S) because each stress level will damage the workpiece at a different number of cycles (N). Then, both values are used to create the S–N graph of the material used and of the stress ratio used. 

## Figures and Tables

**Table 1:** The chemical composition of AA7075-T651 by the energy-dispersive X-ray fluorescence (EDXRF) method.

Elements	Cu	Mn (Max)	Mg	Zn	Cr	Fe (Max)	Al
Standard	1.2–2.0	0.30	2.1–2.9	5.1–6.1	0.18–0.28	0.50	Balance
Measured	1.83	0.09	4.95	7.39	0.32	0.37	Balance

**Table 2:** The mechanical properties of the AA7075-T651 aluminium alloy.

Mechanical Properties	Value
Hardness, Vickers	175
Tensile strength, ultimate	572 MPa
Tensile strength, yield	503 MPa
Modulus of elasticity	71.7 GPa
Poisson’s ratio	0.33
Shear modulus	26.9 GPa
Shear strength	331 MPa
Fatigue strength (5 × 10^8^ cycles)	159 MPa

**Table 3:** Friction stir welding process parameters.

Parameters	Value
Rotational speed	1600 rpm
Welding speed	30 mm/min
Plunge depth	0.1 mm
Plunge feed rate	6 mm/min
Dwell time	15 s
Tool tilt angle	0°

**Table 4:** The deep-rolling process parameters.

Rolling Pressure (Bar)	Rolling Speed (mm/min)	Rolling Offset (mm)
150	1400	0.10

## Abbreviations

AA	Aluminum Alloy
AISI	American Iron and Steel Institute
AS	Advancing Side
ASTM	American Society for Testing and Materials
BM	Base Material
CDRX	Continuous Dynamic Recrystallization
CNC	Computer Numerical Control
DIN	Deutsches Institut für Normung e.V. (German Institute for Standardization)
DR	Deep Rolling
FSW	Friction Stir Welding
FWHM	Full Width at Half Maximum
HV	Vickers Hardness Number
JIS	Japanese Industrial Standards
PWHT	Post-Weld Heat Treatment
RS	Retreating Side
SZ	Stir Zone
TMAZ	Thermo-Mechanically Affected Zone
XRD	X-Ray Diffraction
